# Rare inherited antithrombin deficiency presenting as spontaneous cerebral venous thrombosis with concomitant parenchymal hemorrhage: a case report

**DOI:** 10.3389/fmed.2026.1810172

**Published:** 2026-05-15

**Authors:** Hanlei Zhou, Li Yin, Libin Zhang, Lanting Hu, Weiwei Zhang, Chenyi Ye, Zhenjie Liu

**Affiliations:** 1 Department of Vascular Surgery, The Second Affiliated Hospital, School of Medicine, Zhejiang University, Hangzhou, China; 2 Department of Orthopedics, The Second Affiliated Hospital, School of Medicine, Zhejiang University, Hangzhou, China; 3 State Key Laboratory of Transvascular Implantation Devices, Hangzhou, China; 4 Binjiang Institute of Zhejiang University, Hangzhou, China

**Keywords:** cerebral venous thrombosis, inherited antithrombin deficiency, intracranial hemorrhage, thrombophilia screening, whole-exome sequencing

## Abstract

Inherited antithrombin deficiency (IATD) is a rare thrombophilia that predisposes individuals to venous thromboembolism (VTE), though its presentation as spontaneous cerebral venous thrombosis (CVT) is exceedingly uncommon. We report the case of a 27-year-old woman with IATD who developed CVT complicated by bilateral frontal lobe hemorrhage. The initial manifestation of intracranial hemorrhage posed a significant diagnostic and therapeutic dilemma, as it often contraindicates anticoagulation. This case highlights that in the context of CVT, concomitant hemorrhage should not be considered an absolute contraindication to anticoagulant therapy. Our experience underscores the critical importance of a comprehensive diagnostic workup, including thrombophilia screening, in young patients with unprovoked thrombosis at unusual sites. Furthermore, our experience suggests that direct oral anticoagulants (DOACs) could potentially serve as an effective and safe long-term therapeutic option for individuals with IATD, though this hypothesis requires further validation in larger cohorts.

## 1 Introduction

IATD is an uncommon autosomal dominant disorder that significantly increases the risk of VTE, accounting for only approximately 0.5% of CVT cases due to the rarity of the condition itself (1). Intracranial hemorrhage (ICH) is a severe complication of CVT; major observational cohorts and international multicenter registries have consistently demonstrated that concomitant ICH occurs in approximately 28 to 39% of CVT patients at presentation (2–5). In such settings, anticoagulant treatment is controversial due to the risk of exacerbating hemorrhagic transformation (6). A paucity of literature exists on IATD presenting with CVT and ICH, resulting in limited documented experience in its diagnosis and management. This case report contributes to the limited literature by describing a young female with unprovoked CVT and secondary ICH who was successfully managed through comprehensive thrombophilia screening and a tailored anticoagulation regimen. The advent of direct oral anticoagulants (DOACs) offers a promising investigational option for lifelong anticoagulation in patients with unprovoked CVT and persistent risk factors such as thrombophilia.

## 2 Case description

A 27-year-old female of Asian descent was admitted to a local hospital following a transient loss of consciousness and seizures without apparent precipitating factors. The episode was characterized by generalized tonic–clonic convulsions lasting approximately 2 min before resolving spontaneously. Cerebral computed tomography angiography (CTA) revealed bilateral frontal lobe hemorrhage and ruled out arterial etiologies, but a veno-CT scan (CTV) was not performed. The patient received osmotic therapy with mannitol and anti-seizure medication with levetiracetam; however, the etiology of the cerebral hemorrhage remained undetermined.

On day 11 of hospitalization, the patient complained of swelling and pain in the left lower limb. Doppler ultrasound confirmed deep vein thrombosis (DVT) involving the entire left iliac and femoral veins. The patient denied any history of chronic illness, surgery, recurrent miscarriage, or trauma. Notably, one of her younger brothers presented with left lower limb swelling at the age of 23 and was diagnosed with DVT. He was managed with a standard therapeutic dose of rivaroxaban, achieving complete resolution without recurrence.

**Figure 1:**
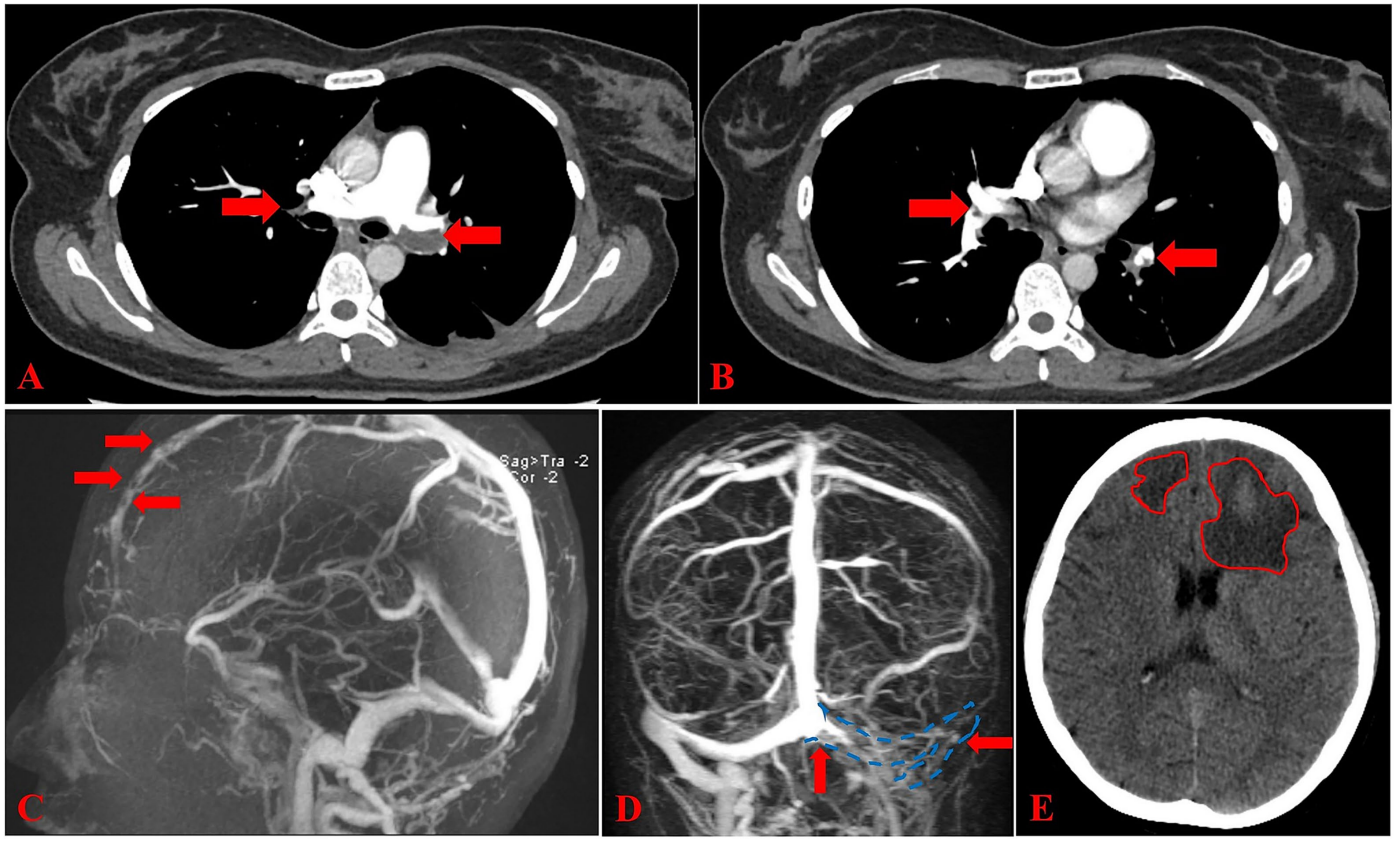
Imaging findings at admission. **(A,B)** CTPA reveals multiple pulmonary artery embolism (arrows). **(C)** Sagittal magnetic resonance venography (MRV) shows thromboembolism in the superior sagittal sinus (arrows). **(D)** Coronal MRV demonstrates thrombotic occlusion in the left transverse sinus and sigmoid sinus (arrows). **(E)** Axial non-contrast cranial CT scan reveals mixed-density shadows in both frontal lobes, consistent with subacute hemorrhage in the absorption phase (outlined by irregular circles).

The patient was subsequently referred to our hospital for further investigation and management. Urgent computed tomography pulmonary angiography (CTPA) upon admission revealed emboli in the distal left main pulmonary artery and its bilateral branches (Figures 1A,B). Magnetic resonance venography (MRV) demonstrated venous thrombosis in the superior sagittal sinus (Figure 1C), left transverse and sigmoid sinus (Figure 1D). In consultation with neurology specialists, the intracranial venous thrombosis was determined to have caused venous rupture and subsequent cerebral hemorrhage. A cranial CT scan demonstrated bilateral frontal hematomas in the absorption phase (Figure 1E). Pretreatment blood tests revealed a plasma dimerized plasmin fragment D (D-dimer) level of 11,250 μg/L (reference<500 μg/L), a platelet count of 217 × 10^9^/L, plasma protein C activity of 99.00% (reference 70–130%), plasma protein S activity of 92.00% (reference 55–123%), and an erythrocyte sedimentation rate of 8 mm/h. Additionally, testing for antiphospholipid antibodies, anticardiolipin antibodies, rheumatoid factor, antinuclear antibodies, and anti-neutrophil cytoplasmic antibodies yielded negative results, thereby excluding common acquired thrombophilias.

Therapeutic anticoagulation was initiated with low molecular weight heparin (LMWH) at a dose of 5,000 IU every 12 h. Four hours after the second dose, the anti-factor Xa level for LMWH was 0.04 IU/mL (therapeutic range 0.5–1.0 IU/mL). Concurrent antithrombin activity was 38% (reference 80–120%), raising suspicion for antithrombin deficiency. However, recognizing that antithrombin levels can be transiently reduced during an acute thrombotic event, a reassessment of these values after the acute disease stage was deemed necessary for a definitive diagnosis. Although we tried to titrate the LMWH dose upward to reach the target range, we remained cautious about potential heparin resistance and dosing reliability due to the low antithrombin activity.

An inferior vena cava (IVC) filter was first implanted to prevent pulmonary embolism during endovascular manipulation. Subsequently, to rapidly reduce the massive thrombus burden and mitigate the long-term risk of post-thrombotic syndrome, mechanical thrombectomy of the left lower extremity deep vein was performed using an AngioJet Thrombectomy System (Boston Scientific, Marlborough, Massachusetts, United States). During the procedure, progressive bradycardia occurred due to a vagal reflex, prompting discontinuation of thrombus aspiration and cancelation of further pulmonary angiography. A thrombolytic catheter was placed from the iliac vein to the femoral vein for catheter-directed thrombolysis. Balancing the risk of the extensive iliac-femoral DVT against the stability of the cerebral hematomas (in the absorption phase), and given the persistent risk of intracerebral hemorrhage, a low dose of urokinase (100,000 IU, twice daily) was administered through the catheter. Concurrently, systemic anticoagulation was utilized with Unfractionated Heparin (UFH) to maintain a safe and effective anticoagulation intensity via continuous monitoring of the activated partial thromboplastin time (APTT). We initiated UFH with a bolus of 80 U/kg followed by an infusion of 18 U/kg/h, and adjusted the infusion rate based on APTT. Due to the patient’s antithrombin deficiency, standard weight-based heparin nomograms failed to achieve therapeutic targets. Therapeutic levels (APTT range: 60–80 s) were ultimately achieved by titrating the infusion rate upward, and the maintenance dose was sustained at 30–40 U/kg/h. Three days later, the thrombolytic catheter was removed. Given the reduced acute thrombus burden and the persistent risk of intracranial re-bleeding, we opted to bypass the standard intensified oral loading dose (15 mg twice daily). Instead, the patient was transitioned directly to a maintenance dose of rivaroxaban 20 mg once daily to minimize peak-concentration-related bleeding risks. The patient’s normal renal function further supported this dosing choice.

**Figure 2:**
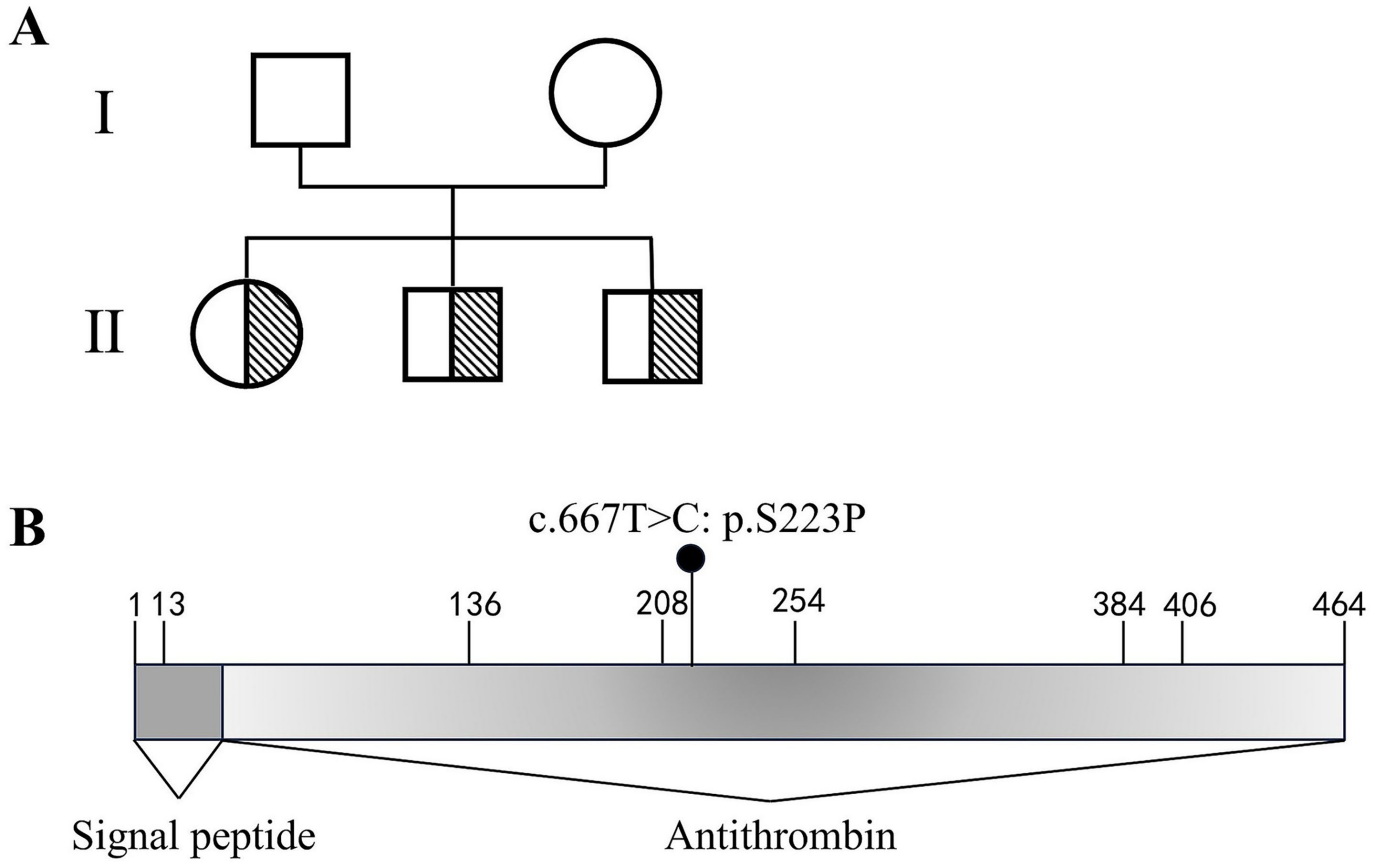
Genetic analysis confirming inherited antithrombin deficiency. **(A)** Pedigree diagrams of the antithrombin deficient family. **(B)** Schematic representation of the *SERPINC1* mutation. A heterozygous c.667 T > C missense variant was identified in exon 4, resulting in a serine-to-proline substitution at amino acid position 223 (p.S223P).

After one additional week of medication, the patient was alert and oriented, with a normal neurological examination. Swelling in the left lower limb had resolved, and no chest tightness or shortness of breath was reported. Given that heparin itself can reduce antithrombin activity, plasma activity was retested 1 week after LMWH discontinuation; it remained significantly below normal levels. Therefore, next-generation whole-exome sequencing (Novogene, Beijing, China) was performed, identifying a pathogenic variant (SERPINC1 c.667 T > C, p.S223P) consistent with IATD. Genetic sequencing of her two younger brothers confirmed the same finding (Figure 2). The patient was discharged with a prescription for long-term oral anticoagulation with rivaroxaban 20 mg once daily.

**Figure 3:**
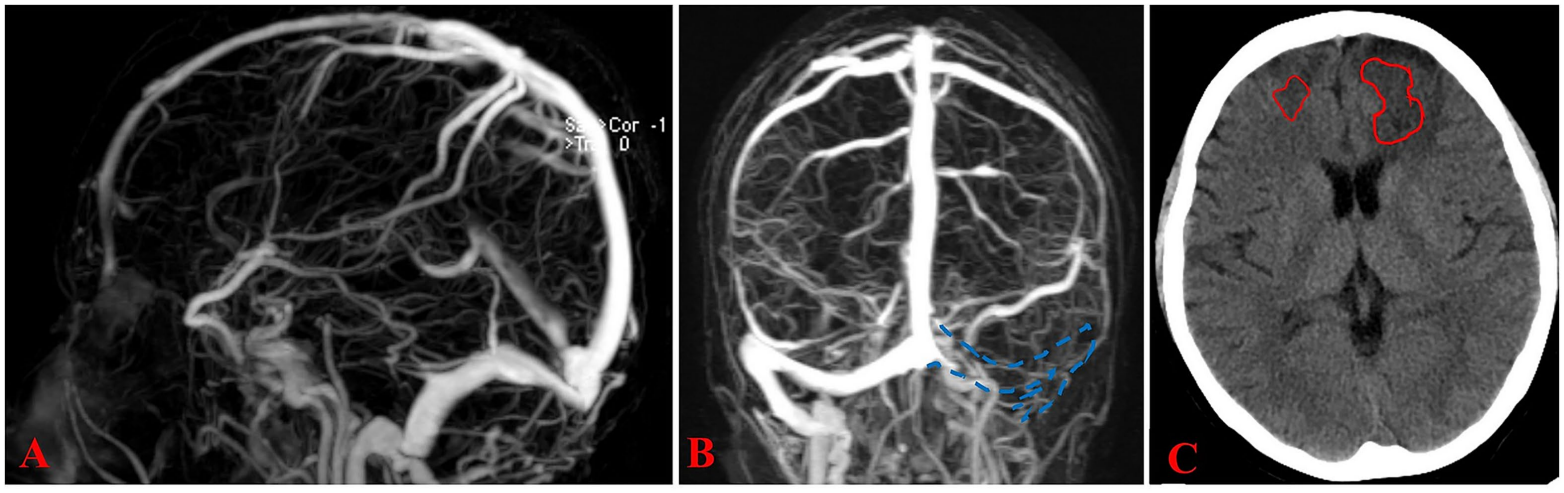
Follow-up imaging at one-month post-discharge. **(A)** Follow-up MRV demonstrates marked resolution of thrombosis within the superior sagittal sinus. **(B)** A residual thrombus is present in the left transverse and sigmoid sinuses, showing interval reduction in size. **(C)** Bilateral frontal hematomas exhibit significant resolution (outlined); no new hemorrhage is evident.

**Figure 4:**
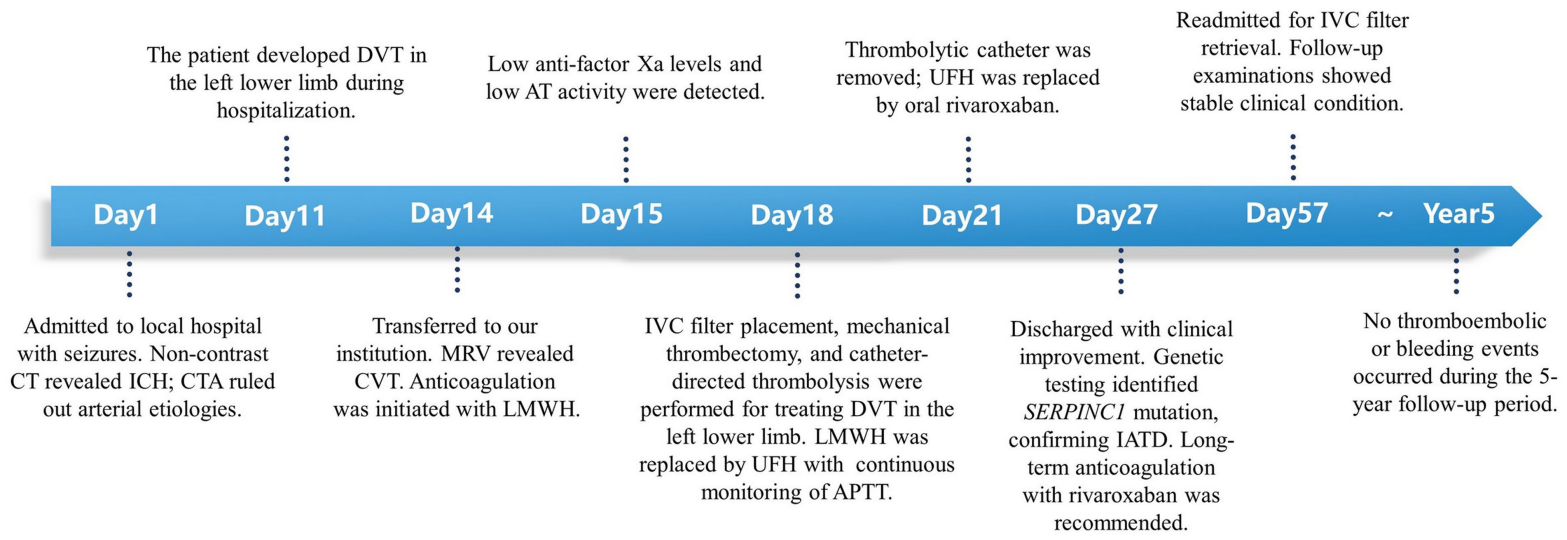
Timeline of the clinical course. The graphic illustrates the patient’s presentation, key diagnostic findings, therapeutic interventions, and follow-up milestones over the 5-year period.

One month later, she was re-admitted for inferior vena cava filter removal. Follow-up testing demonstrated normalized D-dimer levels, improvement in intracranial venous thrombosis (Figures 3A,B), and absorption of cerebral hemorrhage (Figure 3C). The patient and her symptomatic brother were advised to receive lifelong anticoagulation therapy with rivaroxaban 20 mg daily, the asymptomatic younger brother was counseled on thrombosis awareness and advised to receive thromboprophylaxis during high-risk situations (e.g., surgery, trauma, or prolonged immobilization). No thromboembolic or bleeding events occurred during the 5-year follow-up period (Figure 4).

Patient Perspective: Initially, I was terrified by the stroke and brain bleeding at my age. However, the genetic diagnosis of inherited antithrombin deficiency brought relief, explaining the cause and enabling family screening. Transitioning to oral rivaroxaban has significantly improved my daily life, allowing me to live normally without the constant fear of recurrence.

## 3 Discussion

### 3.1 Diagnostic challenges of CVT in atypical demographics

Recent epidemiological studies highlight an increasing incidence of CVT with significant racial disparities; the age- and sex-standardized rates are highest among Black populations (23.1 cases/million) and lowest among Asians (8.56 cases/million) (7). The occurrence of severe CVT in our patient of Asian descent—a demographic with an inherently low baseline risk—is therefore exceptionally rare.

CVT is a rare but potentially life-threatening condition, frequently manifesting with non-specific symptoms such as headache, lethargy, seizure, or altered consciousness, which can pose diagnostic challenges. CVT leads to increased venous pressure and reduced blood flow, causing rupture of veins and capillaries, which subsequently results in parenchymal hemorrhage (8). In patients with cerebral infarctions extending beyond typical arterial territories or with lobar hemorrhages of unknown origin, additional imaging of the cerebral venous system is recommended. MRV is particularly useful for detecting CVT when CT findings are inconclusive (9, 10). The presence of a cord-like area of high signal intensity in the sinus, referred to as the “cord sign,” is a characteristic finding on T1 weighted MRI image (11). In the present case, MRV was pivotal for definitive diagnosis.

### 3.2 Acute anticoagulation strategy in the presence of hemorrhage

According to the guidelines from the American Heart Association/American Stroke Association (AHA/ASA) and the European Stroke Organization (ESO), anticoagulation remains the standard therapeutic approach for acute cerebral venous thrombosis (CVT). Both guidelines explicitly emphasize that baseline hemorrhage does not constitute a contraindication for anticoagulant therapy (10, 12). Recently, Katwal et al. reported a successful outcome in a patient with CVT and hemorrhagic infarction treated with low molecular weight heparin followed by vitamin K antagonists (11). Two randomized controlled trials that included patients with pretreatment cerebral hemorrhage observed no new or worsened hemorrhages following anticoagulation (4, 13). These findings support the hypothesis that anticoagulation improves venous outflow obstruction, thereby reducing venular and capillary pressure and the risk of further hemorrhage (6, 8). In the case described above, navigating the balance between thrombosis control and bleeding risk was the central challenge. The patient required catheter-directed thrombolysis for extensive DVT while simultaneously managing a recent bilateral frontal lobe hemorrhage. Given this precarious scenario, we prioritized the safety profile and controllability of the anticoagulant agent. Consequently, we transitioned from LMWH to UFH because of its shorter half-life, and a dynamic, APTT-guided dose titration strategy was employed. This approach allowed us to successfully overcome the resistance threshold and maintain effective anticoagulation intensity without exacerbating the pre-existing intracranial hemorrhage.

### 3.3 Genetic profiling and heparin resistance in IATD

Identifying the underlying etiology in young patients with CVT is critical for preventing recurrence. CVT is uncommon in the general population but more prevalent among individuals aged <55 years, those with thrombophilia, and pregnant women or those using hormonal contraceptives (8, 10). According to the International Study on Cerebral Vein and Dural Sinus Thrombosis (ISCVT) cohort, thrombophilia was present in 34% of cases, including 22% with inherited forms (4). Testing for inherited thrombophilia remains controversial, with some experts arguing against routine testing because results may not alter VTE management (14). The American Society of Hematology (ASH) conditionally recommends thrombophilia testing in some special scenarios (15).

In the present case, whole-exome sequencing identified a heterozygous missense variant c.667 T > C in exon 4 of the SERPINC1 gene, resulting in a serine-to-proline substitution at amino acid position 223 (p.S223P). This variant corresponds to the S191P mutation in the mature protein numbering system. Its pathogenicity has been well-established in previous studies. Picard et al. identified this mutation in families with recurrent venous thrombosis and classified it as causing Type I antithrombin deficiency. Mechanistically, the residue Ser191 is located on Helix F of the antithrombin molecule; its substitution by Proline is predicted to disrupt the helix’s internal hydrogen bonding and packing, leading to protein instability and impaired secretion (16). Furthermore, a striking genotype–phenotype correlation exists for this variant regarding cerebrovascular involvement. Baud et al. reported a neonate carrying the identical S191P mutation who succumbed to fatal cerebral venous thrombosis complicated by parenchymal hemorrhage (17). The confirmation of this pathogenic variant, which was also detected in the patient’s siblings, underscores the necessity for lifelong anticoagulation. Although genetic testing was not performed on the patient’s minor daughter, genetic counseling regarding VTE risk is strongly recommended upon her reaching adulthood, particularly prior to conception, given the autosomal dominant inheritance pattern of IATD.

IATD is associated with a high lifetime risk of VTE, with an annual absolute risk of a first VTE of approximately 2% and a recurrence risk of up to 55% (18). Inherited thrombophilia represents an unmodifiable, lifelong predisposition to CVT (19). Many guidelines explicitly advise lifelong anticoagulation for individuals with inherited thrombophilia disorders (20–22). Additionally, IATD patients are prone to heparin resistance, as the anticoagulant effect of heparin heavily relies on sufficient antithrombin levels (23, 24). Consequently, standard LMWH or UFH doses often result in under-treatment (25). For suspected heparin resistance due to antithrombin deficiency, a regimen combining antithrombin concentrates and anti–factor Xa-adjusted therapeutic dose LMWH is considered effective (26, 27).

### 3.4 Long-term anticoagulation and the potential role of DOACs

However, for patients requiring long-term anticoagulation, oral agents may represent a more convenient option. DOACs are increasingly replacing vitamin K antagonists. The RE-SPECT CVT randomized clinical trial (RCT) demonstrated that DOACs offer a safety and efficacy profile comparable to dose-adjusted warfarin (28). Specifically, regarding rivaroxaban, the EINSTEIN-Jr CVT RCT provided robust evidence that rivaroxaban is safe and highly effective for treating CVT, showing no symptomatic recurrent VTE and a favorable bleeding profile compared to standard anticoagulation (29). Furthermore, the large multicenter ACTION-CVT study revealed that DOACs, including rivaroxaban, are associated with similar rates of venous recanalization but with a significantly lower risk of major hemorrhage compared to VKAs (30). This efficacy and safety have been most recently corroborated by the prospective international DOAC-CVT cohort study (31). Given our patient’s concomitant intracranial hemorrhage and the clinical need for a stable, predictable anticoagulant effect without frequent laboratory monitoring, rivaroxaban was considered an optimal and evidence-based choice. DOACs directly target and inhibit coagulation factors IIa (thrombin) or Xa and may be effective in individuals with antithrombin deficiency (32). Due to the rarity of this condition, evidence for the use of DOACs primarily derives from case reports (26, 33, 34). Furthermore, recent clinical studies with DOACs in antithrombin deficiency have primarily focused on deep vein thrombosis or pulmonary embolism, with a paucity of data concerning CVT complicated by intracranial hemorrhage (32, 35). Our case helps bridge this gap by suggesting that DOACs might be a safe and effective long-term therapeutic option even in IATD patients presenting with CVT and concomitant hemorrhage, serving as a hypothesis-generating observation for future clinical trials. Despite the successful outcome in this instance, the primary limitation of this report is its nature as a single case, which restricts the generalizability of the findings, alongside the current lack of randomized evidence for this specific clinical scenario.

## 4 Conclusion

This case demonstrates that inherited antithrombin deficiency can first present as CVT with ICH in young patients. Crucially, even when ICH is present, anticoagulation is not strictly contraindicated if CVT is confirmed; rather, it must be administered carefully with close monitoring. Furthermore, genetic testing plays an indispensable role in confirming the diagnosis and guiding long-term treatment strategies. While recent landmark RCTs have established the overall safety and efficacy of DOACs in general CVT populations, their optimal use and timing in patients with severe hereditary thrombophilias remain ongoing clinical questions in stroke unit management. Future multi-center registries are essential to formulate definitive therapeutic protocols for such high-risk subgroups. Although this represents a single case, our patient has demonstrated an excellent clinical trajectory, with no thrombotic recurrences or bleeding complications during a 5-year follow-up. As a single-case observation, this report adds meaningful clinical evidence suggesting the viability of a tailored DOAC strategy in similar complex clinical settings; however, these findings are primarily hypothesis-generating and necessitate further validation in larger, multi-center cohorts.

## Data Availability

The original contributions presented in the study are included in the article/supplementary material. Further inquiries can be directed to the corresponding author.
